# Effects of hospital delivery during off-hours on perinatal outcome in several subgroups: a retrospective cohort study

**DOI:** 10.1186/1471-2393-12-92

**Published:** 2012-09-08

**Authors:** Ronald Gijsen, Chantal WPM Hukkelhoven, C Maarten A Schipper, Uzor C Ogbu, Mieneke de Bruin-Kooistra, Gert P Westert

**Affiliations:** 1 Centre for Public Health Forecasting, National Institute for Public Health and the Environment, PO Box 1, Bilthoven, BA, 3720, The Netherlands; 2 The Netherlands Perinatal Registry, PO Box 8588, Utrecht, RN, 3503, The Netherlands; 3 Expertise Centre for Methodology and Information Services, National Institute for Public Health and the Environment, PO Box 1, Bilthoven, BA, 3720, The Netherlands; 4 RAND Corporation, 1776 Main Street, Santa Monica, CA, 90405, USA; 5 Department of Social Medicine, Academic Medical Center, University of Amsterdam, Amsterdam, The Netherlands; 6 Centre for Health Services Research, National Institute for Public Health and the Environment, PO Box 1, Bilthoven, BA, 3720, The Netherlands; 7 Scientific Institute for Quality of Healthcare (IQ Healthcare), Radboud University Nijmegen Medical Centre, PO Box 9101, Nijmegen, HB, 6500, The Netherlands

**Keywords:** Time of birth, Night, Weekend, Delivery, Perinatal mortality, Perinatal morbidity, Hospital care, Quality of health care

## Abstract

**Background:**

Studies have demonstrated a higher risk of adverse outcomes among infants born or admitted during off-hours, as compared to office hours, leading to questions about quality of care provide during off-hours (weekend, evening or night). We aim to determine the relationship between off-hours delivery and adverse perinatal outcomes for subgroups of hospital births.

**Methods:**

This retrospective cohort study was based on data from the Netherlands Perinatal Registry, a countrywide registry that covers 99% of all hospital births in the Netherlands. Data of 449,714 infants, born at 28 completed weeks or later, in the period 2003 through 2007 were used. Infants with a high a priori risk of morbidity or mortality were excluded. Outcome measures were intrapartum and early neonatal mortality, a low Apgar score (5 minute score of 0–6), and a composite adverse perinatal outcome measure (mortality, low Apgar score, severe birth trauma, admission to a neonatal intensive care unit).

**Results:**

Evening and night-time deliveries that involved induction or augmentation of labour, or an emergency caesarean section, were associated with an increased risk of an adverse perinatal outcome when compared to similar daytime deliveries. Weekend deliveries were not associated with an increased risk when compared to weekday deliveries. It was estimated that each year, between 126 and 141 cases with an adverse perinatal outcomes could be attributed to this evening and night effect. Of these, 21 (15-16%) are intrapartum or early neonatal death. Among the 3100 infants in the study population who experience an adverse outcome each year, death accounted for only 5% (165) of these outcomes.

**Conclusion:**

This study shows that for infants whose mothers require obstetric interventions during labour and delivery, birth in the evening or at night, are at an increased risk of an adverse perinatal outcomes.

## Background

At present, a considerable amount of literature has been published about the relationship between hospital admissions that occur in the evening, at night, or during the weekend, and morbidity and mortality. In obstetrics and neonatal care, studies have focused on the time of birth, or admission to a neonatal intensive care unit (NICU) [1,2]. Studies have demonstrated a higher risk of adverse outcomes among infants born or admitted during off-hours (weekend, evening or night), as compared to office hours, leading to questions about the quality of care provided during off-hours. However, the findings of studies examining the effect of time of birth on perinatal mortality and morbidity have been inconsistent. Some studies reported increased risks for births during the weekend [3-9], during the evening or night [8-24], or during off-hours [8,25,26], while others did not find any effect [10,27-33]. In addition, many studies did not take into consideration that interventions like induction and augmentation of labour, administration of analgesics or anaesthetics, planned and emergency caesarean sections, or instrumental deliveries, are not randomly carried out throughout the day and week, and are directed to high-risk pregnancies.

In this paper, we aim to determine the relationship between off-hours delivery and adverse perinatal outcomes for subgroups of hospital births that require obstetric interventions. Focusing on subgroups may give important insight in specific processes of care. We also estimated the number of adverse outcomes attributable to the off-hours effect, among all births and within the subgroups. The expression of the risk as a number, instead of an odds ratio, may give a better indication of the impact of the off-hours effect on public health, and the potential gains of possible improvements in health care quality.

## Methods

### Data sources

For this retrospective cohort study we used data from the Netherlands Perinatal Registry (PRN). This countrywide registry covers 95% of the approximately 180,000 live-born infants and stillbirths per year in the Netherlands [34]. The PRN is based on a validated probabilistic linkage [35,36] of three voluntary independent registries owned by the professional organizations of midwives, obstetricians, and neonatologists/paediatricians. In 2007, the participation rates of midwife practices, obstetric departments, and paediatric departments were 94%, 99% and 68% respectively [34]. One hundred percent of the paediatric departments with a neonatal intensive care unit participated. Data on the mother, the pregnancy, childbirth, the child, and progress of care are registered using standardized electronic forms. Once a year the data are sent to the national registry office, which performs several quality checks. If necessary, data are sent back to the professionals, who are given ample opportunity to correct them.

### Study population

**Figure 1:**
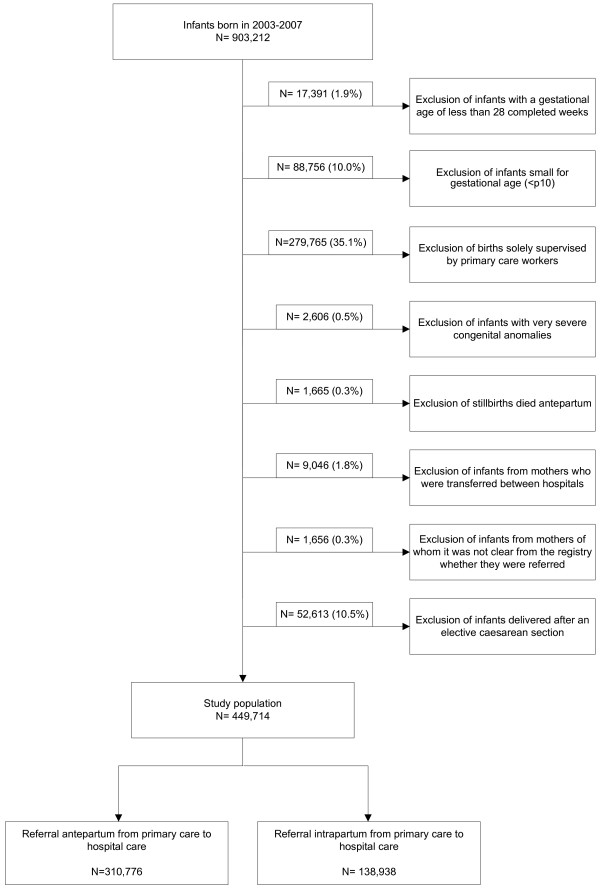
Flowchart showing the selection of infants available for analysis.

Data on infants born in the years 2003 through 2007 were used. In this period, about two-third of births took place in the hospital (supervised by a gynaecologist) and one third at home or in birth centres (supervised by a primary care midwife or general practitioner). Mothers can choose to give birth at home (or in a birth centre) if they have no known risk factors for complications at the onset of delivery. Our study was limited to those births that took place in a hospital. Infants with a high a priori risk of morbidity or mortality were excluded, namely infants at a gestational age below 28 completed weeks, infants small for gestational age (birth weight below the 10^th^ percentile) [37-39], infants with very severe congenital anomalies, and infants born to mothers who were transferred between hospitals during pregnancy or delivery, e.g. from secondary care to tertiary care. Babies who died before the start of delivery (antepartum deaths) were also excluded. Because elective caesarean sections are predominantly done during office hours, and the usual policy in the Netherlands is to perform these only in high-risk pregnancies, they were also excluded. The selection process is represented in Figure 1.

We distinguished infants born to mothers referred from primary to secondary or tertiary care during labour or delivery (‘intrapartum transfer to the hospital’), from infants born to mothers who were already under hospital care before the onset of labour (‘antepartum transfer to the hospital’ or ‘referred before the onset of labour’). The latter group consists of both women who were under secondary care from the beginning of pregnancy, and women referred to secondary care during pregnancy before the onset of labour. The two groups, intrapartum and antepartum transfer to the hospital, may have different baseline risks and additional risks (as a consequence of a referral process under a certain level of urgency). We excluded birth records of women for whom it was unclear whether they were referred.

### Outcome variables

We used three dichotomous outcome variables: (1) intrapartum and early neonatal mortality (death of the unborn child during labour or delivery, and death within 7 days after live birth, respectively), (2) a low Apgar score (5 minute score of 0–6), and (3) a composite measure. The composite measure combined intrapartum and early neonatal mortality, a low Apgar score, severe birth trauma (excluding cephalic haematoma, fracture of the clavicle, facial nerve injury and injury to the brachial plexus) [40], and admission to a NICU on the same or the day after birth.

### Time of birth

Time of birth was examined using three different categorizations. The first was based on the day of the week and defined as weekday (Monday 8:00 am till Friday 10:59 pm) versus weekend (Friday 11:00 pm till Monday 7:59, and national holidays), the second was based on the time of the day and defined as day (from 8:00 am till 5:59 pm), evening (from 6:00 pm till 10:59 pm), and night (from 11:00 pm till 7:59 am). The third was based on an aggregation of the day of the week and time of the day into off-hours (evening, night, or the weekend) and office hours (daytime during weekdays).

### Case-mix variables

We distinguished between two types of case-mix variables, namely (1) socio-biological factors and (2) characteristics of the delivery and obstetric interventions performed. Socio-biological factors included birth weight (in grams), gestational age at delivery (28–36, 37–41, ≥42 completed weeks), congenital anomalies (infants with very severe anomalies were excluded; remaining anomalies were divided into mild and severe, based on perinatal mortality risks of congenital anomalies [41], fetal position (cephalic, breech, or transverse/other position), general medical or obstetric problems of the mother (as recorded by the midwife or gynaecologist), maternal age (<25, 25–34, ≥35 years), parity (0, 1, ≥2), single/multiple pregnancies, sex of the child, ethnicity of the mother (western, non-western countries), socioeconomic status (low, average, high), and the degree of urbanization of the maternal place of residence (5 classes). The characteristics of the delivery and obstetric interventions performed included induction and/or augmentation of labour (yes, no), administration of analgesics or anaesthetics (none/light analgesics, opiates, epidural anaesthesia in the first stage of labour, epidural/spinal anaesthesia during caesarean section, general anaesthesia), mode of delivery (spontaneous delivery, instrumental vaginal delivery - vacuum or forceps extraction -, emergency caesarean section), hospital type (tertiary referral centre with a NICU, teaching hospital without a NICU, general hospital without a NICU), year of delivery, and the duration of the second stage of labour (categorized as 0–29, 30–59, 60–119, ≥120 minutes). However, the start time of interventions is not registered in the PRN.

### Statistical analyses

To analyse the outcome measures, we performed multilevel logistic regression analyses controlling for case-mix differences between infants born during the different time periods used in the categorization of time of birth. Multilevel models account for potential clustering of adverse perinatal outcomes within hospitals. They also correct for systematic differences between hospitals in the potential association between time of birth and outcome.

All models included separate variables for time of the day and day of the week. This enabled us to study the effect of each factor separately by adjusting for the other one.

Based on the literature, we included case-mix variables expected to influence perinatal outcome or the association between time of birth and perinatal outcome in the analyses. Observations with missing values on any of these variables were excluded from the data. For each subgroup, our baseline model consisted of time of birth and socio-biological factors. Subsequent models were extended by including the characteristics of the delivery and the obstetric interventions performed.

Observational studies are prone to ‘confounding by indication’. In this case, it refers to the situation in which a determinant of adverse perinatal outcome is an indication for stimulating delivery during a certain part of the day or week. Induction or augmentation of labour and caesarean section in particular, are means to influence the time of birth of a high-risk pregnancy. To minimize this kind of bias, we performed subgroup analyses, with subgroups defined by induction and/or augmentation of labour, combined with the mode of delivery (spontaneous delivery, instrumental delivery, emergency caesarean section). We expected that the risk of adverse perinatal outcomes and their distribution during the day and week would differ between these subgroups, and that within these subgroups confounding by indication would be negligible. We combined subgroups, in which the association between time of birth and outcome did not differ (tested with interaction terms of time of birth and mode of delivery).

The occurrence of the outcome intrapartum and early neonatal mortality was rare. Therefore, only a limited number of potential confounders could be included in the models with mortality as outcome variable. We included those variables that had a p-value <0.05 for testing the association in the corresponding models with the composite measure. Additional, variables were removed through a stepwise backward selection procedure, using restricted likelihood ratio tests (critical p-value at 0.10).

The strength of the association between time of birth and outcomes are expressed as odds ratios (OR) with 95% confidence intervals (CI). To adjust for multiple comparisons, we used the adjustment method of Holm, as explained by Aickin and Gensler [42]. The risk models were also used to calculate the number of cases with an adverse perinatal outcome, attributable to the off-hours effect [43]. Theoretically, this is the reduction in adverse perinatal outcomes that would be observed if the off-hours effect could be eliminated. In this scenario infants born during off-hours have the same risk of an adverse perinatal outcome as infants born during office hours.

The statistical analyses were carried out using SAS version 9.2 [44]. The multilevel logistic regression analysis was done with the SAS procedure GLIMMIX.

## Results

After applying our exclusion criteria (Figure 1), 449,714 infants were included in the study population. Most infants were eliminated because they were born outside the hospital. Of the study population, 310,776 (69%) were referred to a hospital before the onset of labour and 138,938 (31%) were referred during labour.

Table 1 shows the distribution of births by time of referral and time of birth, and the occurrence of the adverse perinatal outcomes in each subgroup. Among infants born to mothers referred to a hospital before the onset of labour, differences were observed in the prevalence of all three adverse outcomes among all three time categorizations (off-hours vs. office hours; day of the week; time of the day). Among infants born to mothers referred to a hospital during labour, differences were observed in the prevalence of a low Apgar score, and the composite measure within the time categorizations off-hours vs. office hours, and time of the day, but not for day of the week.

**Table 1:** Distribution of infants by time of referral and time of birth, and the occurrence of the adverse perinatal outcomes

	**Births, n (%)**	**Intrapartum and early neonatal mortality (%)**	**Apgar score 0–6 (%)**	**Composite outcome (%)**^**†**^
**Referred to a hospital antepartum (n =310776)**				
Office hours	123689 (39.8)	0.14*	1.08*	3.24*
Off-hours	187087 (60.2)	0.21	1.46	4.32
Weekdays	224380 (72.2)	0.18*	1.28*	3.73*
Weekends	86396 (27.8)	0.21	1.39	4.32
Day	158496 (51.0)	0.15*	1.10*	3.37*
Evening	64952 (20.9)	0.20	1.58	4.21
Night	87328 (28.1)	0.24	1.49	4.61
**Referred to a hospital intrapartum (n =138938)**				
Office hours	46266 (33.3)	0.17	1.15*	2.41*
Off-hours	92672 (66.7)	0.19	1.31	2.76
Weekdays	92255 (66.4)	0.18	1.26	2.59
Weekends	46683 (33.6)	0.19	1.24	2.75
Day	65023 (46.8)	0.17	1.12*	2.45*
Evening	28760 (20.7)	0.20	1.34	2.70
Night	45155 (32.5)	0.19	1.39	2.90

^*^ p value for testing differences between time of birth groups with χ^2^-tests < 0.05.

^†^ Composite outcome consists of intrapartum and early neonatal mortality, Apgar score of 0–6, severe birth trauma, and admission to an neonatal intensive care unit.

Tables 2 and 3 show the results of the analyses using the multivariate models. Because the differences between the results of the analyses using the baseline models and the analyses using the extended models were small, we will only describe the results of the extended models. An increased risk of an adverse perinatal outcome was observed among infants born during the evening or night irrespective of whether the mother was under the care of a hospital before the onset of labour or referred during labour or delivery.

**Table 2:** Adjusted odds ratios (95% confidence intervals) for the effect of time of birth by referral status (intrapartum and antepartum) and outcome

***Subgroups***					***Odds ratio (95% CI)***		
					**Time of the day (reference group is day)**		**Part of the week (reference group is weekday)**
**Referral**	**Induction / augmentation**	**Mode of delivery**	**Covariates in model (see notes)**	**Number of infants**	**Evening**	**Night**	**Weekend**
**Intrapartum and early neonatal mortality**							
antepartum	no	spont. / instr.	c	116884	0.86 (0.56-1.33)	0.97 (0.71-1.33)	1.10 (0.81-1.48)
antepartum	no	emergency CS	d	16850	1.15 (0.61-2.17)	1.86 (1.16-2.99)	1.41 (0.92-2.18)
antepartum	yes	all modes	c	174310	1.44 (1.08-1.91)	1.75 (1.32-2.33) ^*^	1.05 (0.81-1.37)
intrapartum	no	spont. / instr.	e	68325	1.17 (0.70-1.96)	1.07 (0.70-1.63)	0.95 (0.64-1.42)
intrapartum	no	emergency CS	f	6806	1.54 (0.73-3.21)	1.70 (0.91-3.18)	1.07 (0.61-1.89)
intrapartum	yes	all modes	f	62913	0.99 (0.58-1.66)	0.84 (0.50-1.44)	1.05 (0.66-1.66)
**Apgar score 0-6**							
antepartum	no	all modes	b	133797	1.06 (0.92-1.23)	1.02 (0.91-1.15)	1.06 (0.95-1.18)
antepartum	yes	spontaneous	b	116571	1.53 (1.31-1.78) ^*^	1.72 (1.47-2.02) ^*^	0.93 (0.80-1.08)
antepartum	yes	instrumental	b	28913	1.44 (1.20-1.73) ^*^	1.43 (1.19-1.73) ^*^	1.06 (0.89-1.26)
antepartum	yes	emergency CS	b	28602	1.02 (0.86-1.21)	1.19 (0.99-1.43)	1.04 (0.88-1.22)
intrapartum	no	spont. / instr.	b	68320	1.22 (1.00-1.50)	1.06 (0.89-1.26)	0.83 (0.71-0.98)
intrapartum	no	emergency CS	a	6805	1.20 (0.84-1.71)	1.40 (1.04-1.87)	1.08 (0.82-1.42)
intrapartum	yes	all modes	a	62908	1.14 (0.95-1.36)	1.38 (1.17-1.63) ^*^	1.00 (0.86-1.16)
**Adverse perinatal outcome (composite measure)**							
antepartum	no	all modes	b	133887	1.01 (0.94-1.10)	0.98 (0.92-1.05)	1.03 (0.97-1.10)
antepartum	yes	spontaneous	b	116593	1.26 (1.15-1.39) ^*^	1.51 (1.37-1.66) ^*^	0.95 (0.86-1.04)
antepartum	yes	instrumental	b	28920	1.16 (1.01-1.33)	1.30 (1.13-1.49) ^*^	1.04 (0.92-1.18)
antepartum	yes	emergency CS	b	28638	1.02 (0.90-1.15)	1.03 (0.90-1.18)	1.05 (0.93-1.18)
intrapartum	no	spont. / instr.	b	68325	1.16 (1.01-1.32)	1.01 (0.90-1.12)	0.97 (0.87-1.08)
intrapartum	no	emergency CS	a	6806	0.97 (0.72-1.32)	1.29 (1.02-1.63)	1.06 (0.85-1.33)
intrapartum	yes	all modes	b	62913	1.05 (0.92-1.20)	1.26 (1.11-1.42) ^*^	0.99 (0.89-1.11)

Models fitted with both time of the day and day of the week, and socio-biological factors.

^*^significant association (< 0.05) after using the Holm correction method for adjusting for multiple comparisons.

^a^birth weight, gestational age at delivery, congenital anomalies, foetal head position, general medical or obstetric problems of the mother, maternal age, parity, sex, ethnicity of the mother, socioeconomic status, degree of urbanization of the maternal place of residence.

^b^(a) + single/multiple pregnancies.

^c^birth weight, gestational age at delivery, congenital anomalies, foetal head position, ethnicity of the mother, sex.

^d^birth weight, single/multiple pregnancies.

^e^birth weight, congenital anomalies, foetal head position, parity.

^f^birth weight, gestational age at delivery, congenital anomalies, foetal head position.

**Table 3:** Adjusted odds ratios (95% confidence intervals) for the effect of time of birth by referral status (intrapartum and antepartum) and outcome

***Subgroups***					***Odds ratio (95% CI)***		
					**Time of the day (reference group is day)**		**Part of the week (reference group is weekday)**
**Referral**	**Induction / augmentation**	**Mode of delivery**	**Model covariates (see notes)**	**Number of infants**	**Evening**	**Night**	**Weekend**
**Intrapartum and early neonatal mortality**							
Antepartum	No	Spont. / instr.	c	116884	0.86 (0.56-1.33)	0.99 (0.72-1.35)	1.10 (0.81-1.49)
Antepartum	No	Emergency CS	j	16850	1.14 (0.60-2.16)	1.84 (1.14-2.96)	1.41 (0.92-2.17)
Antepartum	Yes	All modes	e	174151	1.43 (1.07-1.90)	1.78 (1.35-2.40) ^*^	1.08 (0.82-1.41)
Intrapartum	No	Spont. / instr.	a	68325	1.16 (0.69-1.93)	1.05 (0.69-1.61)	0.95 (0.63-1.42)
Intrapartum	No	Emergency CS	b	6806	1.47 (0.69-3.12)	1.48 (0.78-2.83)	1.05 (0.58-1.89)
Intrapartum	Yes	All modes	b	62913	0.90 (0.53-1.53)	0.79 (0.46-1.35)	1.06 (0.67-1.67)
**Apgar score 0-6**							
Antepartum	No	All modes	f	133650	1.08 (0.93-1.25)	1.08 (0.96-1.21)	1.05 (0.95-1.17)
Antepartum	Yes	Spontaneous	g	116358	1.34 (1.15-1.56) ^*^	1.54 (1.31-1.80) ^*^	0.93 (0.80-1.08)
Antepartum	Yes	Instrumental	g	28848	1.41 (1.17-1.69) ^*^	1.42 (1.17-1.72) ^*^	1.06 (0.89-1.26)
Antepartum	Yes	Emergency CS	f	28602	1.08 (0.90-1.28)	1.17 (0.98-1.42)	1.08 (0.92-1.28)
Intrapartum	No	Spont. / instr.	i	68265	1.23 (1.00-1.50)	1.07 (0.90-1.27)	0.83 (0.71-0.98)
Intrapartum	No	Emergency CS	d	6805	1.18 (0.82-1.70)	1.30 (0.97-1.76)	1.10 (0.83-1.45)
Intrapartum	Yes	All modes	k	62865	1.06 (0.89-1.28)	1.29 (1.09-1.52)	1.00 (0.86-1.17)
**Adverse perinatal outcome (composite measure)**							
Antepartum	No	All modes	f	133734	1.00 (0.92-1.09)	1.00 (0.94-1.07)	1.03 (0.97-1.10)
Antepartum	Yes	Spontaneous	g	116380	1.24 (1.13-1.37) ^*^	1.43 (1.29-1.58) ^*^	0.92 (0.84-1.01)
Antepartum	Yes	Instrumental	g	28855	1.20 (1.04-1.37)	1.24 (1.07-1.42)	1.00 (0.88-1.14)
Antepartum	Yes	Emergency CS	f	28638	1.03 (0.91-1.17)	1.02 (0.89-1.18)	1.07 (0.95-1.21)
Intrapartum	No	Spont. / instr.	i	68270	1.17 (1.02-1.34)	1.02 (0.91-1.14)	0.98 (0.89-1.09)
Intrapartum	No	Emergency CS	d	6806	0.96 (0.70-1.30)	1.21 (0.95-1.54)	1.04 (0.83-1.30)
Intrapartum	Yes	All modes	h	62870	0.96 (0.84-1.11)	1.16 (1.03-1.32)	1.00 (0.89-1.12)

Models fitted with both time of the day and day of the week, and socio-biological factors and characteristics of the delivery and obstetric interventions performed.

^*^significant association (< 0.05) after using the Holm correction method for adjusting for multiple comparisons.

^a^birth weight + congenital anomalies + foetal head position + administration of analgesics or anaesthetics + parity.

^b^birth weight + congenital anomalies + foetal head position + administration of analgesics or anaesthetics + gestational age at delivery.

^c^(b) + sex + ethnicity of the mother + hospital type + mode of delivery (spontaneous, instrumental, emergency section).

^d^(b) + general medical or obstetric problems of the mother + maternal age + parity + sex + socioeconomic status + degree of urbanization of the maternal place of residence + hospital type + year of birth + ethnicity of the mother.

^e^(c) + year of birth.

^f^(d) + single/multiple pregnancies.

^g^(f) + duration of the second stage of labour.

^h^(f) + mode of delivery (spontaneous, instrumental, emergency section).

^i^(g) + mode of delivery (spontaneous, instrumental, emergency section).

^j^birth weight + single/multiple pregnancies + hospital type.

^k^(d) + ethnicity of the mother + mode of delivery (spontaneous, instrumental, emergency section).

The risk of intrapartum or early neonatal death for infants born to mothers who were referred before the onset of labour is increased if labour was induced or augmented, and birth took place in the evening or at night. It was also increased if labour occurred naturally and delivery was performed by emergency caesarean section at night. Infants born in the evening or at night had an increased risk of a low Apgar score if their mothers were referred before the onset of labour, had labour induced or augmented, and achieved spontaneous or instrumental vaginal delivery. Irrespective of the mode of delivery, children of mothers referred during labour and in who labour was augmented were more likely to have a low Apgar score if the delivery occurred during the night. Increased risk of the composite outcome during evening or night birth was observed among subgroups similar to those at risk of a low Apgar score.

Among children born to mothers who were referred before the onset of labour, had no induction or augmentation of labour, and achieved spontaneous or instrumental delivery, we observed no increase in risk associated with birth during the evening, at night orduring the weekend. Weekend birth was not associated with an increased risk in an adverse perinatal outcome for any subgroup when compared to weekday births.

After adjusting for multiple comparisons, using the Holm correction method, half of the findings remained significant at the 0.05 level (see Tables 2 and 3).

Table 4 shows the number of cases that can be attributed to the off-hours effect in the period 2003 through 2007, assuming that the effect measures as calculated and presented in Tables 2 and 3, are true. The predicted number of cases with an adverse perinatal outcome attributable to the off-hours effect is between 630 and 704, depending on the statistical model used. This represents 4% to 4.5% of all cases with adverse perinatal outcomes. The majority (64-67%) of these infants were born to mothers who were already under the care of the hospital before the onset of labour, whose labour was induced or augmented, and who achieved vaginal (spontaneous or instrumental) delivery. The number of cases of intrapartum and early neonatal death attributable to the off-hours effect is 103, in both models. Of these, 59 to 65% were under hospital care before the onset of labour and labour was induced or augmented. The population attributable risk (PAR) for this subgroup is over 20%. A substantial proportion (18-32%) of perinatal deaths among infants born to mothers who were neither induced nor augmented, and who eventually delivered by an emergency caesarean section is attributable to the off-hours effect, irrespective of referral before or during labour. For a low Apgar score and the composite outcome measure the picture is similar, although the PARs for a low Apgar score are somewhat higher.

**Table 4:** Calculated number of cases attributable to the off-hours effect per subgroup and in total in the years 2003 through 2007

			***Models with only social-biological factors***				***Models with social-biological factors, characteristics of the delivery and obstetric interventions performed***			
**Subgroups**			**Number of infants with adverse perinatal outcome**				**Number of infants with adverse perinatal outcome**			
**Referral**	**Induction/ augementation**	**Mode of delivery**^**a**^	**Observed**	**Expected with elimination of off-hours effect**	**Attributable to off-hours effect**	**PAR**^**a**^**(in %)**	**Observed**	**Expected with elimination of off-hours effect**	**Attributable to off-hours effect**	**PAR**^**a**^**(in %)**
**Intrapartum and early neonatal mortality**											
Antepartum	No	Spont. / instr.	187	188	−1	−0.4	187	186	1	0.3	
Antepartum	No	Emergency CS	88	60	28	32.2	88	60	28	32.3	
Antepartum	Yes	All modes	298	237	61	20.5	297	230	67	22.5	
Intrapartum	No	Spont. / instr.	112	108	4	3.2	112	109	3	2.5	
Intrapartum	No	Emergency CS	53	40	13	25.1	53	43	10	18.2	
Intrapartum	Yes	All modes	86	88	−2	−2.9	86	91	−5	−6.2	
*Total*			*824*	*721*	*103*	*12.5*	*823*	*720*	*103*	*12.5*	
**Apgar score 0-6**											
Antepartum	No	All modes	1525	1466	59	3.9	1520	1432	88	5.8	
Antepartum	Yes	Spontaneous	1030	844	186	18.0	1027	888	139	13.5	
Antepartum	Yes	Instrumental	698	569	129	18.5	697	573	124	17.8	
Antepartum	Yes	Emergency CS	776	734	42	5.4	776	717	59	7.6	
Intrapartum	No	Spont. / instr.	688	689	−1	−0.1	688	684	4	0.5	
Intrapartum	No	Emergency CS	249	210	39	15.7	249	216	33	13.4	
Intrapartum	Yes	All modes	791	698	93	11.8	790	721	69	8.8	
*Total*			*5757*	*5209*	*548*	*9.5*	*5747*	*5230*	*517*	*9.0*	
**Adverse perinatal outcome (composite measure)**											
Antepartum	No	All modes	5919	5892	27	0.4	5903	5844	59	1.0	
Antepartum	Yes	Spontaneous	3078	2746	332	10.8	3065	2783	282	9.2	
Antepartum	Yes	Instrumental	1394	1251	143	10.3	1387	1264	123	8.9	
Antepartum	Yes	Emergency CS	1553	1519	34	2.2	1553	1508	45	2.9	
Intrapartum	No	Spont. / instr.	1762	1733	29	1.7	1757	1712	45	2.6	
Intrapartum	No	Emergency CS	420	383	37	8.8	420	395	25	5.9	
Intrapartum	Yes	All modes	1465	1362	103	7.0	1464	1414	50	3.4	
*Total*			*15591*	*14887*	*704*	*4.5*	*15549*	*14919*	*630*	*4.0*	

^a^ abbreviations: *spont.* = spontaneous, *intr.* = instrumental, *CS* = caesarean section, *PAR* = population attributable risk.

## Discussion

### Results in perspective

Birth in the hospital in the evening or at night was associated with an increased risk of perinatal morbidity and/or mortality. These risks were concentrated in subgroups of deliveries that involved induction or augmentation of labour, or an emergency caesarean section. Infants born during off-hours to mothers referred before the onset of labour, whose labour was not induced and augmented, and who achieved vaginal delivery (spontaneous or instrumental) were not at increased risk of an adverse perinatal outcome. Birth during the weekend was not associated with an increased risk of adverse perinatal outcomes for any subgroup.

The PAR calculations demonstrated that about 4 to 4.5% of all cases with an adverse perinatal outcome, and 12.5% of all cases of intrapartum and early neonatal mortality, can be attributed to the evening and night effect. This theoretically established figure can be interpreted as the proportion of adverse outcomes that could be reduced by eliminating the off-hours effect, on the condition that unmeasured confounding does not bias the off-hours effects. Our PAR calculation of perinatal mortality is comparable with calculations from Sweden (12%) and Scotland (16.5%) [8,13]. Health care quality improvement programs could target subgroups with both large absolute and relative numbers of cases that are attributable to the off-hours effect. In this case, it may be worthwhile to focus on deliveries among mothers referred before the onset of labour and whose labour is induced or augmented. Women under hospital care before the onset of labour, who are not induced or augmented, and who need an emergency caesarean section are the second largest contributor to the off-hours effect as it relates to intrapartum or early neonatal mortality.

The increased risks observed among infants born during the evening and night, confirm the results of those other studies accounting for the mode of delivery and/or several other risk factors [8,9,11-16,18-22,24]. Three studies that adjust for several risk factors did not find an evening or night effect. However, all three studies were carried out in tertiary hospitals with round the clock in-house physicians [27,29,30].

In our study, the adjusted odds ratios for birth in the weekend did not differ from 1. This is comparable to the results of most other studies that took mode of delivery and/or other risk factors into account [13,14,21,24,28,29]. Three exceptions were a study among teenage mothers [3] and two studies in which the outcome measure was perinatal mortality due to asphyxia [7,8]. In one study an increased risk of perinatal mortality was demonstrated for infants born in nontertiary hospitals on Saturday. Also an increased risk of adverse perinatal outcome for infants born in tertiary hospitals on Saturday was found. Other combinations of type of hospital, day of the weekend, and outcome measure did not reach significance [9].

### Methodological considerations

This observational study was carried out using a nationwide registry that included nearly all hospital births in the Netherlands. A limitation of observational studies is the sensitivity to ‘confounding by indication’. We minimized this effect by analysing subgroups of infants, defined on the basis of obstetric interventions. Moreover, we sought to compose a homogeneous group of cases, by excluding infants with a high a priori probability of an adverse perinatal outcome. This selection may limit the generalizability of the results, but prevents bias of strong confounding variables. Finally, in our analysis we included random effects for hospitals and adjusted for a large number of potentially confounding factors.

As with most observational studies there is the possibility of the presence of unmeasured confounding. One such factor may be the duration of the first stage of labour. Babies born during the evening or the night may have been exposed to a longer first stage of labour, and consequently have a higher risk of an adverse outcome. The duration of the first stage cannot be determined from the PRN, since the time of the onset of labour is not registered. In clinical practice this is also often omitted. Another potentially confounding factor among the subgroup of infants born to mothers who were referred before the onset of labour, may be the distinction between induction and augmentation. Induction of labour is often started for medical reasons, while augmentation is generally administered to mothers with a prolonged delivery after a spontaneous start. The perinatal risks can be different between these groups. In addition, in contrast to augmentation, induction is often planned, so the time of birth is more controlled. To obtain more insight into the off-hours effects within these subgroups, it may be interesting to differentiate between women who are induced and women who are augmented. However, the reliability of a distinction between the two obstetric interventions in a perinatal registry has to be ascertained.

We cannot rule out that some cases are misclassified, for example in the other caesarean section class (planned or emergency) or time of death class (antepartum death or intrapartum death). In a recent study, some of the cases initially classified as antepartum death, were reclassified as intrapartum deaths after review by a multidisciplinary team [45]. A mild underreporting of early neonatal mortality is expected, since one third of the paediatric departments in Dutch hospitals did not participate in the PRN at the time of our study. Furthermore, midwives, gynaecologists and paediatricians can make mistakes when entering the data. Some of these may remain undetected by the national registry office when checking the data. Finally, some cases had missing values on the examined variables, although, the number of cases with missing data was very limited (0.9% of the study population).

After adjustment for multiple comparisons, half of the associations remained significant. However, the discussion about the need to adjust for multiple comparisons is not yet settled [46]. Therefore, those associations that did not remain significant after adjustment for multiple comparisons may still represent a true relationship between the time of delivery and adverse outcomes.

### Possible explanations of the associations

The off-hours effect convincingly demonstrated in our study may be caused by a delayed recognition of perinatal risks in the evening or at night, and an inappropriate response to hazardous situations. This may be the result of a multiple factors, like diminished numbers of and expertise of staff available, reduced access to diagnostic tests and procedures, a lower degree of supervision of residents, long-duration shifts and tiredness of personnel, no in-house obstetricians, anaesthesiologists and paediatricians, delays in availability of necessary personnel in case of emergency. In the Netherlands, the round-the-clock in-house presence of an obstetrician, anaesthesiologists, and the operating room team, is not warranted in the majority of the hospitals. Despite speculation about the impact of all these factors [47], they have not been extensively studied.

In our study, we did not demonstrate an increased risk of adverse perinatal outcomes among the subgroup of mothers who were referred before the onset of labour, whose labour was not induced or augmented, and who achieved vaginal delivery (spontaneously or instrumental). This suggests that for this subgroup differences in quality of obstetric care or other risk factors between birth during off-hours and daytime did not play an important role.

The absence of a weekend effect found in this study suggests that the quality of care during daytime, evening and night during the weekend does not differ from corresponding parts of the day during weekdays. Despite the reduced staffing numbers in the weekend, during the daytime the available health personnel may be alert enough to prevent and reduce hazardous situations. Delays in availability of personnel, who are on duty during the weekend, may be comparable to those during corresponding parts of the day during weekdays.

## Conclusion

Although confounding in our study cannot be entirely excluded, we recommend that the quality and organization of perinatal care should be optimized for the identified risk groups during the evening and the night, irrespective of how the causal pathway leads to adverse outcomes. This off-hours effect has also been demonstrated in other countries. Because we focused on hospital births, excluding home births which are rare in other countries, we think our results may be generalizable to other countries. A next step in research may be the identification of the factors that lead to the increased risks and an examination of the risks to infants not included in this study (like small for gestational age infants).

## Competing interests

The authors declare that they have no competing interests.

## Authors’ contributions

RG, CWPMH, CMAS, MBK, and GPW were primarily responsible for developing the study protocol. RG and CWPMH extracted the data and RG performed the analyses. CMAS advised on the statistical analyses. All authors participated in interpretation of the results. RG wrote the first draft of the paper and all authors contributed in the revision of the manuscript and approved the final version of the paper. All authors have critically read and approved the final manuscript.

## Pre-publication history

The pre-publication history for this paper can be accessed here:

http://www.biomedcentral.com/1471-2393/12/92/prepub
